# Crop Diversity at the Landscape Level Affects the Composition and Structure of the Vegetation-Dwelling Arthropod Communities in Naked Oat (*Avena Chinensis*) Fields

**DOI:** 10.3390/ijerph18010030

**Published:** 2020-12-23

**Authors:** Huan Zhao, Jiahuan Li, Lizhu Guo, Kun Wang

**Affiliations:** 1 College of Grassland Science and Technology, China Agricultural University, Beijing 100193, China; zhaohuan333@163.com (H.Z.); lijhecol@163.com (J.L.); ellenguo@sina.cn (L.G.); 2 GuYuan National Grassland Ecosystem Field Station, Zhangjiakou 076550, China

**Keywords:** planting pattern, crop diversity, arthropod community, farmland landscape, natural enemy, herbivorous pests, biological pest control

## Abstract

The expansion of agriculture and intensive mechanized production have resulted in the loss of habitats and biodiversity, which has led to the loss of ecological services such as the biological control of pests and diseases, and insect-borne pollination. Current studies mainly focus on the impact of small-scale crop diversity (such as intercropping) on ecological service but lack research on the effects of crop diversity at the landscape scale. In this study, vegetation-dwelling arthropods in naked oat (*Avena chinensis*) fields under different planting patterns were collected at different growth stages by standardized sweep netting sampling, and the differences in arthropod communities and temporal dynamics were analyzed. Taking this information as an example, the effects of crop diversity at the landscape scale caused by different planting patterns on arthropod communities were studied. It was found that herbivores were the most abundant functional group in the arthropod community in naked oat fields, accounting for 70.13% of the total abundance, followed by natural enemies, accounting for 23.45%, and, finally, other insects. The abundance and species richness of natural enemies in naked oat fields under diversified planting pattern were significantly higher than those under intensive planting pattern, while the abundance and species richness of herbivorous pests showed no significant difference between the two planting patterns. Planting patterns significantly affected the composition and structure of arthropod communities in naked oat fields. Significantly higher ratio of natural enemy to pest and more diverse natural enemies under the diversified planting pattern have shown better biological control potential and the significance of biodiversity protection.

## 1. Introduction

Agricultural land is the most important land use mode on the earth, accounting for 40% of the surface of nonfrozen land and providing an important source of food for human survival [1]. With the improvement of mechanization, intensive and homogeneous agricultural areas are becoming increasingly common in order to produce more food [2]. Intensive agricultural production leads to simplification of the farmland landscape, and the compositional and configurational heterogeneity of the farmland landscape decreases. Specifically, the proportion occupied by crop monocultures are increasing in size, and the variation in types of crops and number of natural and semi-natural habitats are decreasing [3]. Numerous studies [2,4,5,6,7] have shown that agricultural intensification results in the reduction of natural enemies’ abundance and species richness, thus leading to the reduction of associated ecological services such as biological pest control, and the increased use of pesticides [8]. The loss of these ecosystem services is often thought to be related to the decline in biodiversity, and, over the past half century, the expansion and intensification of agriculture has led to the loss of habitats and biodiversity [1,3].

In agricultural landscapes, habitats are mainly divided into crop habitats and non-crop habitats [4]. Non-crop habitats mainly refer to natural and semi-natural habitats. The abundance of natural enemies is higher in landscapes with higher proportion of non-crop habitats as they provide food resources, improve the microclimate, and provide overwintering habits to improve the ecological services of biological pest control [9,10,11]. However, a high proportion of non-crop habitats do not always provide more natural enemies to control the abundance of pests in some cases [6]. Tscharntke et al. [12] proposed five hypotheses to show that natural habitats are not necessarily improving the biological control of pests. However, there is no doubt that non-crop habitats are conducive to the heterogeneity of the agricultural landscape. Crop habitats are also an important component of the compositional heterogeneity of the agricultural landscape [3]. Crop diversity, whether at the field level or at the territory level, can reduce production inputs (water, fertilizers, pesticides, etc.), improve the heterogeneity of habitat patches, stabilize yields, and improve the sustainability of the farmland system [13,14]. Intercropping, as an important cropping system for crop diversity, increases diversity at the field scale by planting at least two crops on the same land at the same time. Many studies show that intercropping improves the soil quality, controls weeds, increases the crop yield and stability, and improves the utilization of environmental resources (light, water, nutrients, etc.) during crop growth [15,16,17]. As an important component of biodiversity, crop diversity has been suggested to be effective in controlling pests on a small spatial scale [18,19]. Secondary crops in crop habitats, although small in size, also play a significant role in agricultural ecosystems, providing alternative hosts for the pests of main crops as well as alternative prey, nectar resources, shelters, and overwintering habitats for natural enemies.

Increasing attention has been paid to the management of farmland landscape habitats, which is considered to be an effective way to increase the abundance of natural enemies and, thus, support important ecosystem services such as biological pest control [20]. Pest biocontrol can reduce the use of chemical pesticides and increase production [21]. To protect and optimize the ecosystem services of biological pest control in agricultural systems, it is necessary to understand the relationship between pests and their natural enemies at the local and landscape scales and the corresponding impact of landscape heterogeneity at different scales [21].

At present, most studies on landscape heterogeneity focus on the effects of overall landscape heterogeneity (including all land use types) or on the effects of the proportion of natural and semi-natural habitats [22,23,24], while there are fewer studies on crop habitat heterogeneity [25]. Research on the effects of crop habitat heterogeneity on pest control is also often concentrated at the small scale (field scale), such as in field-scale intercropping experiments [15,19,26]. The impact of farmland landscape heterogeneity on biological pest control has been shown to be scale-dependent [4]. At the landscape scale, we do not know how crop diversity affects biological pest control still lacks research [2,19]. Compared with small-scale crop diversity in the field, crop diversity at the landscape scale is of greater practical significance to farmers, because it is more convenient in agricultural operations such as sowing, field management, and harvesting. In addition, existing research focuses more on a few important arthropods [11,21,27,28], and the temporal dynamics of arthropod communities are rarely studied [9].

Therefore, the effects of crop diversity at the landscape scale on the composition and structure of vegetation-dwelling arthropod communities in naked oat (*Avena chinensis*) fields (a very important crop in the northern agro-pastoral ecotone) caused by different planting patterns were studied as research examples in order to explore the impact of crop diversity on the vegetation-dwelling arthropod communities at the landscape scale. In addition, the dynamic changes of vegetation-dwelling arthropod communities in naked oat fields during different growth stages were studied to explore the changes of arthropod communities in different growth stages of naked oat. Ultimately, management suggestions and theoretical support are expected to be provided for biodiversity conservation and sustainable agricultural development.

## 2. Materials and Methods

### 2.1. Study Area

**Figure 1:**
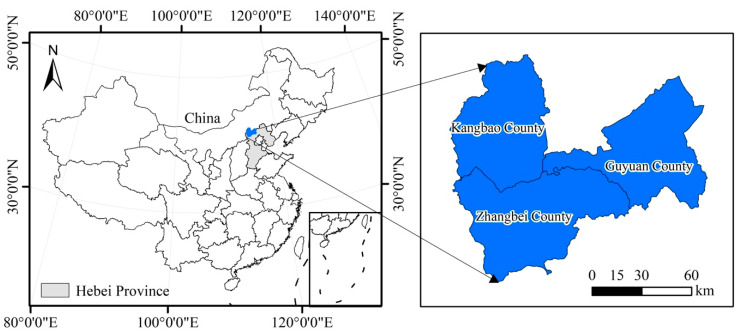
Map showing the location of the study area.

This research was carried out in three counties (Guyuan County, Zhangbei County, and Kangbao County) of Zhangjiakou City, Hebei Province, China in 2018 (Figure 1). The climate of this region is characterized by a temperate continental climate. The temperature of the study area at the time of sampling was 293–303 K. The annual average temperature is approximately 275 K, and the annual average precipitation is approximately 400 mm. The precipitation is mainly concentrated in June, July, and August, which are the hottest months of the year. The altitude of this region is approximately 1400 m.

**Figure 2:**
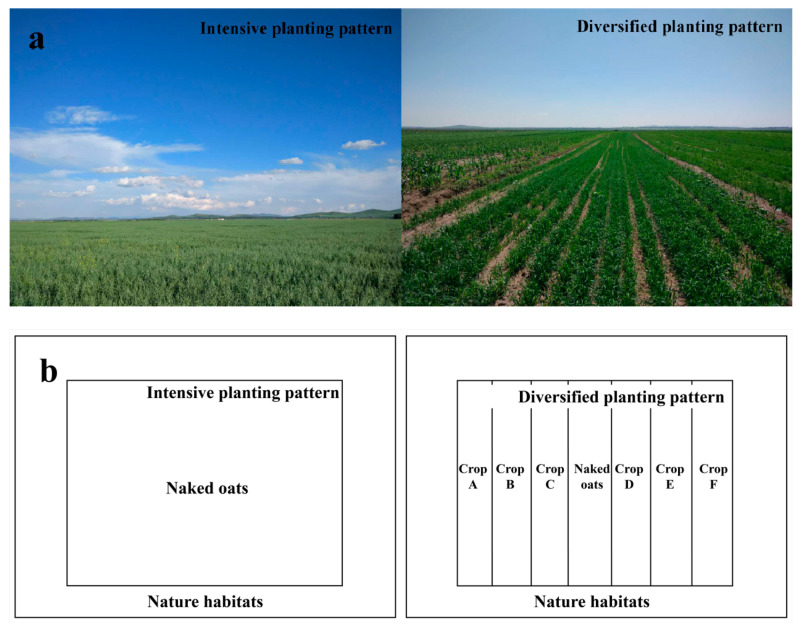
Schematic figures showing naked oat under different planting patterns: (**a**) pictures of naked oat fields; and (**b**) schematic diagrams of naked oat fields.

The study area belongs to the agro-pastoral transitional zone of Northern China. Animal breeding and agriculture coexist. The crops planted in this area are mainly naked oat, flax (*Sesamum indicum*), silage corn (*Zea mays*), potato (*Solanum tuberosum*), cabbage (*Brassica oleracea*), etc. The planting pattern of agriculture mainly includes the diversified planting pattern and intensive planting pattern. The former is a small-scale peasant economy planting model with a long history, with each family planting a small area of farmland separately. The latter is a modern intensive mechanized large-scale single crop planting pattern. Some farmers in this region have transferred farmland to enterprises, and enterprises have centralized and unified the management of large areas of land for agricultural production, forming an intensive planting pattern. Some farmers have retained their own farmland and planted different crops according to their needs, forming a diversified planting pattern. The farmland with different crops forms different landscape units. The main crop of this region, naked oat, is present in both planting patterns (Figure 2). The shorter side or the width of single-planted naked oat land is usually greater than 100 m (Figure 2a), and the shorter side of diversified naked oat land is usually approximately 10 m (Figure 2b). The longer side of both of them is about 200 m. There are significant differences in crop diversity between the two planting patterns at the landscape level. There are on average 12.83 ± 0.93 crop types under the diversified planting pattern, but only one under the intensive planting pattern.

### 2.2. Sampling Methods

In Guyuan County, Zhangbei County, and Kangbao County in the study area, 4, 3, and 3 naked oat fields under intensive planting pattern and 4, 3, and 3 naked oat fields under diversified planting pattern were selected, respectively. All farmland sample plots had similar field management practices, and no pesticides were used. The distances between the sample plots were not less than 1 km.

The arthropods on the naked oat were collected using standardized sweep netting sampling, which is a sampling method that can effectively collect arthropods inhabiting the vegetation and allow explore the response of arthropods to plant diversity [29,30]. Each sample plot was sampled in 100 sweeps according to a “Z” shape in the farmland. The sampling rate was kept constant at approximately 0.5 m/s. The insect net used had a rod length of 1 m, a mesh opening diameter of 35 cm, a mesh depth of 60 cm, and a mesh diameter of 0.15 mm. The arthropods captured were first anesthetized with diethyl ether. Then, the anesthetized arthropods were put into a 50 mL centrifugal tube, 75% medical alcohol was added for storage, and the samples were brought back to the laboratory for identification. Most arthropods were identified at the family level. Because the arthropod larvae and adults were captured together, and the larvae were difficult to identify in terms of genus, the arthropods were identified at the family level or even the order level [31]. Thus, we measured family richness as a proxy for species richness. In Guyuan County, the plots were sampled at the tillering stage, jointing stage, and flowering stage of the naked oat. The sample plots in Zhangbei County and Kangbao County were sampled once at the jointing stage of the naked oat.

Arthropods were classified into three functional groups (herbivores, natural enemies, and other insects) according to their feeding habits. Arthropods were also classified into winged and wingless according to their flying ability.

### 2.3. Data Analysis

Community structure indices were calculated based on species individual data. The Shannon–Wiener diversity index (*H’*) was calculated as *H’* = −$\sum P_{i}lnP_{i}$. *P_i_* is the proportion of the *i*th species in the community and ln is the natural log [15,32].

The effects of the planting pattern on the composition and structure of the vegetation-dwelling arthropod communities in naked oat fields were tested with generalized linear mixed-effects models (GLMMs) and linear mixed-effects models (LMMs). The abundance and species richness of vegetation-dwelling arthropods in naked oat fields under different planting patterns were analyzed with Poisson GLMMs because this distribution is usually applied to count data [33]. The community structure indices of the vegetation-dwelling arthropods in naked oat fields under different planting patterns were analyzed with LMMs. Planting patterns were used as explanatory variables, and the sampling site and sampling time were used as random factors in all models. The assumptions of all models were tested according to the residual plots, residual normality, and independence. Only the effects of different planting patterns on the abundance and species richness of vegetation-dwelling arthropods in naked oat fields were analyzed using the survey data of all three counties. The other data analysis in this paper used the subdatasets of arthropods in naked oat fields at different growth stages in Guyuan County, because the investigation in Guyuan County ran through the important time points of naked oat growth.

Differences in the vegetation-dwelling arthropod community in naked oat fields under different planting patterns were determined by conducting a one-way permutational multivariate analysis of variance (PERMANOVA) using Bray–Curtis dissimilarity matrices. A non-metric multidimensional scaling (NMDS) graph was drawn to visually investigate the differences between arthropod communities in naked oat fields under different planting patterns more intuitively. Differences in the abundance and the percentage of different functional groups between two planting patterns and among three growth stages were tested, respectively, by applying one-way ANOVAs, followed by Tukey’s honestly significant difference post hoc test.

All data presented in this paper were analyzed using R version 3.6.0 (R Foundation for Statistical Computing, Vienna, Austria, 2019), with the “lme4” package for the GLMMs and LMMs, the “vegan” package for the NMDS, the “multcomp” package for the ANOVAs, the “car” package for testing the validity of all the models, and the “ggplot2” package for plotting all the statistical charts.

## 3. Results

### 3.1. Composition of the Vegetation-Dwelling Arthropods in Naked Oat Fields over a Single Growing Season

In the three growth stages of naked oat in Guyuan County, 5330 vegetation-dwelling arthropods were collected spanning 2 classes, 12 orders, and 62 families in naked oat fields. The abundance of Insecta accounted for more than 95% of the population. The Diptera in the Insecta occupied the largest number of both species and individuals, including 25 families and accounting for 31.74% of the total arthropods. Aphididae, Thripidae, Agromyzidae, Chrysomelidae, and Syrphidae were the five families with the largest abundance in the arthropod community, accounting for 59.27% of the total arthropods. In terms of functional groups, there were 23 families of herbivores, accounting for 70.13% of the total arthropods, which was the main component of the arthropod community in naked oat fields. In additional, there were 25 families of natural enemies, accounting for 23.45% of the total arthropods. The main natural enemies were Syrphidae, Araneae, Asilidae, Chrysopidae, Braconidae, and Ichneumonidae. Other insects comprised 14 families, accounting for 6.42% of the total arthropods.

### 3.2. Effects of Crop Diversity Caused by Different Planting Patterns on the Arthropod Abundance and Species Richness of Different Functional Groups of Vegetation-Dwelling Arthropod Communities

The analysis results of the GLMMs showed that the crop diversity caused by the different planting patterns had an effect on the abundance and the species richness (Table 1) in the vegetation-dwelling arthropod communities in naked oat fields. There were significant differences in both the abundance and the species richness under different planting patterns. The naked oat field under the diversified planting pattern had significantly higher abundance (*p* < 0.001) and more species (*p* < 0.001) than did that under the intensive planting pattern. When looking at each of functional groups, the abundance of natural enemies and other insects under the diversified planting pattern were significantly higher than those under the intensive planting pattern (*p* < 0.05). However, there was no significant difference in the abundance of herbivores (*p* = 0.298). Similar to the results for the abundance of different functional groups, the species richness of natural enemies and other insects of the vegetation-dwelling arthropod communities in naked oat fields under the diversified planting pattern was significantly higher than that under the intensive planting pattern (*p* < 0.05). Similarly, there was no significant difference in the species richness of herbivores (*p* = 0.071).

### 3.3. Effects of Crop Diversity Caused by Different Planting Patterns on the Arthropod Community Composition and Community Structure Indices in Naked Oat Fields

**Figure 3:**
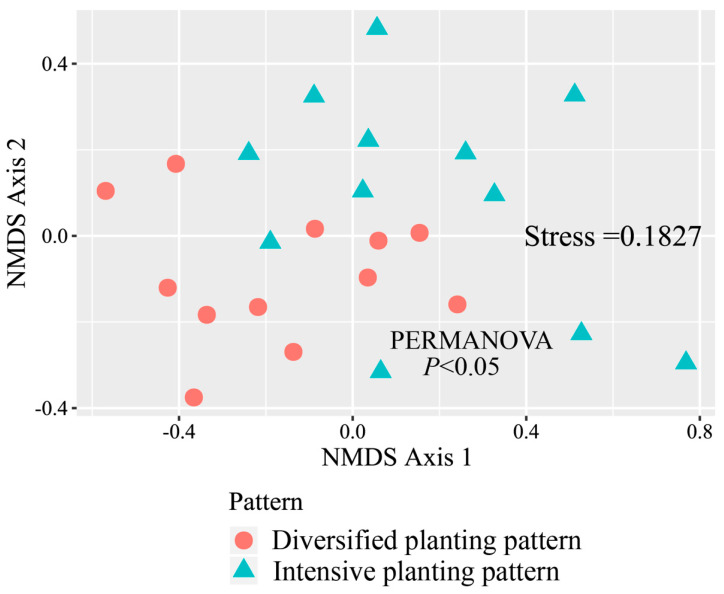
The non-metric multidimensional scaling (NMDS) of community dissimilarity (Bray–Curtis) showing differences in the community compositions of vegetation-dwelling arthropods in naked oat fields under different planting patterns.

The plot of the NMDS analysis showed intuitively that the compositions of the vegetation-dwelling arthropod communities in naked oat fields under different planting patterns were different (Figure 3). At the same time, the results of the PERMANOVA indicated that the arthropod community compositions were significantly different between the two planting patterns (*p* < 0.05).

**Figure 4:**
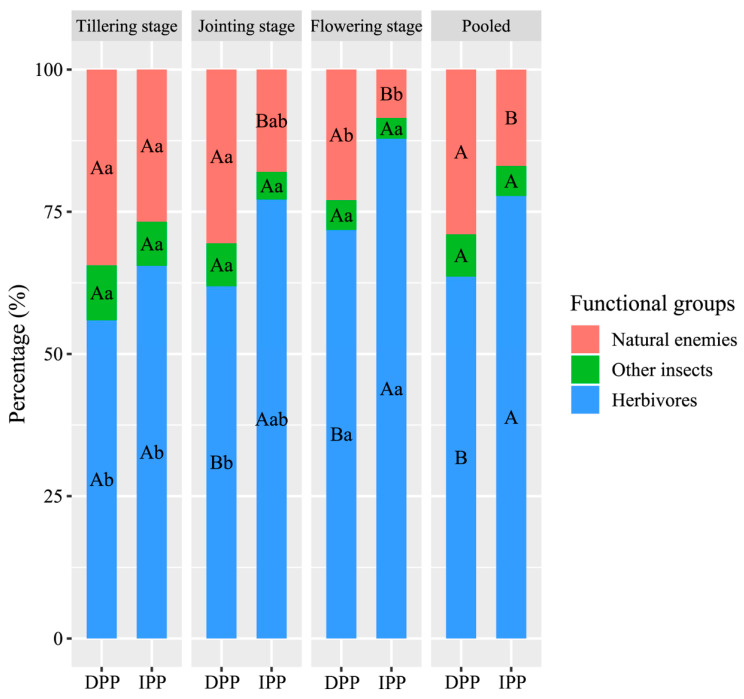
Percentages of different functional groups at different growth stages and under different planting patterns. Values followed by different capital letters are significantly different between different planting patterns (*p* < 0.05). Values followed by different lowercase letters are significantly different among growth stages (*p* < 0.05). DPP, diversified planting pattern; IPP, intensive planting pattern.

In terms of the proportions of different functional groups of vegetation-dwelling arthropods in naked oat fields, the herbivores occupied the greatest proportion, followed by the natural enemies, and, finally, the other insects (Figure 4). Herbivores showed an increasing trend throughout the growth cycle under different planting patterns, while natural enemies and other insects showed the opposite trend. Other insects did not differ significantly between the different planting patterns or among the different growth stages. Natural enemies were significantly higher in the tillering stage than in the flowering stage under the two different planting patterns (*p* < 0.05). In contrast, the proportion of herbivores was significantly higher in the flowering stage than in the tillering stage (*p* < 0.05). Comparisons between different planting patterns revealed that there were no significant differences between natural enemies and herbivores under the different planting patterns in the tillering stage. In the jointing and flowering stages, natural enemies under the diversified planting pattern were significantly higher than those under the intensive planting pattern, while the trend of herbivores was the opposite (*p* < 0.05). In total, the comparisons between different planting patterns were the same as that of the jointing and flowering stages. In addition, the vegetation-dwelling arthropod communities under the diversified planting pattern had a significantly higher ratio of natural enemy to pest than those under the intensive planting pattern (*p* < 0.05, Table 2).

The Shannon–Wiener diversity index can reflect the community structure of an arthropods community [15]. The analysis results of the LMMs showed that the crop diversity caused by the different planting patterns had an effect on this community structure index (Table 2) of the vegetation-dwelling arthropod communities in naked oat fields. The naked oat field under the diversified planting pattern had a significantly greater diversity index than did that under the intensive planting pattern (*p* < 0.001).

### 3.4. Temporal Dynamics of the Abundance of Different Functional Groups of Vegetation-Dwelling Arthropods

**Figure 5:**
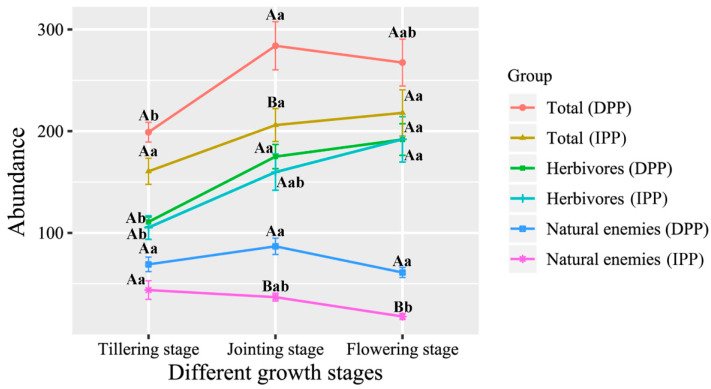
Abundance of different functional groups at different growth stages and under different planting patterns. The error bars indicate the standard errors (SE) of the means. Values followed by different capital letters are significantly different between different planting patterns (*p* < 0.05). Values followed by different lowercase letters are significantly different among growth stages (*p* < 0.05). DPP, diversified planting pattern; IPP, intensive planting pattern.

The temporal dynamics of the abundance of the whole vegetation-dwelling arthropod community and the different functional groups in naked oat fields in three growth stages under the two planting patterns were analyzed (Figure 5). The results show that the abundance of total arthropods was the lowest in the tillering stage under both planting patterns. The abundance of total arthropods in the tillering stage was significantly lower than that in the jointing stage under the diversified planting pattern (*p* < 0.05). However, under the intensive planting pattern, the abundance did not differ significantly among the three growth stages. In addition, the abundance of total arthropods under the diversified planting pattern was significantly higher than that under the intensive planting pattern in the jointing stage (*p* < 0.05). In terms of the abundance of arthropods in different functional groups, the abundance of herbivores was the lowest in the tillering stage, followed by the jointing stage, and the highest in the flowering stage under both planting patterns. The abundance in the flowering stage was significantly higher than that in the tillering stage (*p* < 0.05). There was no significant difference in the abundance of herbivores under the different planting patterns over the three growth stages. Unlike herbivores, the abundance of natural enemies showed a downward trend among the three growth stages. The abundance in the tillering stage was significantly higher than that in the flowering stage under the intensive planting pattern (*p* < 0.05). Furthermore, the abundance of natural enemies under the diversified planting pattern was significantly higher than that under the intensive planting pattern in both the jointing stage and the flowering stage (*p* < 0.05).

## 4. Discussion

From the analysis of the composition of the vegetation-dwelling arthropod communities in naked oat fields, we show that the arthropod communities were mainly dominated by a few dominant species. In addition, the herbivores were the main components of the arthropod community, and abundance was more than twice the abundance of natural enemies and other insects. This is similar to the results of the arthropod community survey in the grassland biodiversity experiment at the Cedar Creek Ecosystem Science Reserve [34], where herbivores occupied the vast majority of the community.

### 4.1. Effects of Crop Diversity at the Landscape Level on the Composition of the Arthropod Communities

The agricultural landscape structure and habitat type have different effects on different trophic levels of arthropod food webs in crops. In the same community, the higher is the trophic level of the species, the larger is the niche it occupies. Conversely, the lower is the trophic level, the smaller is the niche. Species that occupy a larger niche are more affected by habitats [21,35]. In our study, natural enemies at the high trophic level were more sensitive to habitat diversity than were those at the low trophic level, confirming the above assertions. The abundance and species richness of natural enemies in naked oat fields under diversified planting pattern were significantly higher than those under intensive planting pattern (*p* < 0.05), while there was no significant difference in the abundance and species richness of herbivores of low trophic level between the two planting patterns (*p* > 0.05). 

The abundance of natural enemies determines the effectiveness of biological control, and the species diversity of natural enemies is linked to the stability of this ecological service [2]. The higher crop diversity with more and more diverse natural enemies caused by diversified planting pattern may improve the biological control of pests. Secondary crops, in addition to the naked oat in our research, also play an important role in the agricultural landscape. As important habitats for some natural enemies, secondary crop habitats can improve the diversity of natural enemies in the agricultural landscape and allow migration when pest outbreaks occur near farmland [2]. At the same time, secondary crops also create physical and chemical barriers that prevent pests from finding host plants [4]. In addition, a wide variety of crop habitats also offers a wide variety of herbivores that support natural enemies directly or indirectly through the food webs and increases the abundance and species richness of natural enemies in the main crop fields [15]. These assertions support our results: that is, there are more abundant and higher species richness vegetation-dwelling arthropods in naked oat fields under the diversified planting pattern with high crop diversity. Furthermore, the greater abundance of vegetation-dwelling arthropods in naked oat fields under diversified planting pattern mainly consists of winged arthropods, because our study found that winged arthropods in naked oat fields under diversified planting pattern were significantly higher than those under intensive planting pattern (*p* < 0.001, Table 1), while wingless arthropods showed no significant difference between the two planting patterns (*p* = 0.557, Table 1). The response of arthropods to the spatial scale is related to their radius of movement and migration ability [4,6,27]. At the landscape scale we studied, winged arthropods with strong migration ability showed significant differences under different planting patterns.

### 4.2. Effects of Crop Diversity at the Landscape Level on the Structure of the Arthropod Communities

The results of the NMDS in our study were similar to those of Liu et al.’s [15] field-scale intercropping experiment in plots: that is, different crop diversities led to different arthropod communities. However, unlike their findings, high crop diversity farmlands under diversified planting pattern had more abundance and higher species richness of natural enemies and herbivores. There are more types of crop habitats in farmlands under diversified planting pattern. Different types of crops vary in terms of height and density, providing different microenvironments (temperature, light, etc.). Arthropods are poikilotherms, and their metabolism and behavioral activities are sensitive to minor changes in the ambient temperature [15,36]; therefore, farmland landscapes with richer habitats have higher species richness and more diverse arthropod communities. Moreover, the results of the diversity index indicate that the arthropod communities of naked oat under diversified planting pattern had higher diversity and uniformity, while the arthropod community of naked oat under intensive planting pattern had a simpler structure.

### 4.3. Role of Crop Diversity at the Landscape Level

The ecological balance of pests and natural enemies is the key factor in determining the biological pest control effect. A diverse and abundant natural enemy community supports higher pest control [15,37,38]. Concerning the mechanism of natural enemy control, the two hypotheses proposed by Root [39] are widely recognized. One is the resource concentration hypothesis, and the other is the natural enemy hypothesis. The resource concentration hypothesis refers to the fact that herbivorous insects are more inclined to choose a higher-density or single-host plant habitat, which is a bottom-up control approach. The natural enemy hypothesis refers to the fact that diverse habitats can increase the population of natural enemies and thus control pests more effectively, which is a top-down control approach [10,39]. 

In this study, more and more diverse natural enemies under the diversified planting pattern with more heterogeneous landscapes and more diverse habitat types is more conducive to pest control [11,40]. However, simplified landscapes and homogeneous habitats are beneficial to pests and increase the risk of pest outbreaks [7]. There was no significant difference in the abundance of herbivores between the two planting patterns, and the diversified planting pattern did not show an immediate better biological control effect. This may be because the pests in the study area are not serious and farmers generally do not need to use pesticides. Thus, natural enemies play a limited role in pest control. Nevertheless, the significantly higher ratio of natural enemy to pest and more diverse natural enemies under the diversified planting pattern have shown better biological control potential and the significance of biodiversity protection.

## 5. Conclusions

In the agro-pastoral transitional zone of Northern China, the diversified planting pattern of the traditional small-scale peasant economy coexists with the modern mechanized intensive planting pattern, which provides a convenient condition for us to study the effects of crop diversity on arthropod communities at the landscape scale. This study complements the gaps in the large-scale study of the response of arthropod communities to crop diversity. Our study found that there were significant differences in arthropod community compositions between the two planting patterns, and the landscape-scale crop diversity caused by a diversified planting pattern could significantly increase the overall abundance and species richness of the arthropod community in farmland. Among these attributes, the abundance and species richness of natural enemies in naked oat fields under diversified planting pattern were significantly higher than those of naked oat under intensive planting pattern, while herbivorous pests did not differ significantly between the two planting patterns. Moreover, the differences in community composition between the two planting patterns were mainly caused by winged arthropods with a larger active radius and stronger migration ability. At different growth stages, the abundance of natural enemies in naked oat fields under diversified planting structure was higher than that of naked oat under intensive planting pattern, and the growth trend of herbivorous pests was also smaller than that under intensive planting pattern. Moreover, significantly higher ratio of natural enemy to pest and more diverse natural enemies under the diversified planting pattern have shown better biological control potential and the significance of biodiversity protection. 

Compared with the ecological service value exhibited by the crop diversity at the farmland scale, the improved biological pest control effect produced by crop diversity at the landscape scale is of greater significance to farmers. Because the realization of crop diversity at the landscape scale does not require complex habitat management, agricultural activities are simpler, and there are no complicated changes to the methods of sowing, fertilization, and harvesting. It is only necessary to reduce each farmland to a certain extent and increase the types of crops planted at the landscape scale. If the plan is thorough, the workload of farmers will not increase, but a good biological pest control effect will be achieved.

## Figures and Tables

**Table 1:** Statistical results of generalized linear mixed-effects models (GLMMs) for the abundance and richness of vegetation-dwelling arthropods in naked oat fields under different planting patterns. Significant *p*-values are printed in bold.

Response Variables	Metrics	Explanatory Variables	Generalized Linear Mixed-Effects Models			
			Estimate	Std. Error	z value	Pr (>|z|)
Total arthropods	Abundance	Intercept	5.477	0.097	56.389	<0.001
		Intensive planting pattern	−0.236	0.070	−3.374	**<0.001**
	Richness	Intercept	3.178	−0.346	56.715	<0.001
		Intensive planting pattern	−0.346	0.075	−4.642	**<0.001**
Natural enemies	Abundance	Intercept	4.115	0.154	26.652	<0.001
		Intensive planting pattern	−0.711	0.152	−4.686	**<0.001**
	Richness	Intercept	2.120	0.082	25.968	<0.001
		Intensive planting pattern	−0.426	0.130	−3.277	**0.001**
Herbivores	Abundance	Intercept	5.029	0.147	34.113	<0.001
		Intensive planting pattern	−0.062	0.060	−1.041	0.298
	Richness	Intercept	2.378	0.072	33.115	<0.001
		Intensive planting pattern	−0.193	0.107	−1.804	0.071
Other insects	Abundance	Intercept	3.004	0.155	19.428	<0.001
		Intensive planting pattern	−0.549	0.180	−3.041	**0.002**
	Richness	Intercept	1.609	0.105	15.268	<0.001
		Intensive planting pattern	−0.608	0.178	−3.425	**<0.001**
Winged arthropods	Abundance	Intercept	5.200	0.072	72.649	<0.001
		Intensive planting pattern	−0.269	0.076	−3.526	**<0.001**
Wingless arthropods	Abundance	Intercept	3.930	0.253	15.551	<0.001
		Intensive planting pattern	−0.082	0.140	−0.587	0.557

**Table 2:** Statistical results of linear mixed-effects models (LMMs) for the community structure indices of vegetation-dwelling arthropods in naked oat fields under different planting patterns. Significant *p*-values are printed in bold.

Response Variables	Explanatory Variables	Linear Mixed-Effects Models				
		Estimate	Std. Error	t Value	Chisq	Pr (>Chisq)
Diversity index	Intercept	2.646	0.095	27.741	16.268	**<0.001**
	Intensive planting pattern	−0.332	0.082	−4.033		
Ratio of natural enemy to pest	Intercept	0.439	0.100	4.397	20.022	**<0.001**
	Intensive planting pattern	−0.185	0.041	−4.475		
